# Clinical care processes for early postpartum haemorrhage diagnosis: a nested observational study within the E-MOTIVE trial

**DOI:** 10.3389/fgwh.2025.1527261

**Published:** 2025-11-24

**Authors:** Kristie-Marie Mammoliti, Fernando Althabe, Christina Easter, James Martin, Adam J. Devall, Adeosun Love Funmi, Rahmatu Yusuf, Fatima Abubakar, Lolade Christiana Arigbede, JimKelly Mugambi, Polycarp Oyoo, Masumbuko Sambusa, Akwinata Banda, Fawzia Samuels, Sara Willemse, Sibongile Doris Khambule, Hilal Mukhtar Shu'aib, Aminu Ado Wakili, Jenipher Okore, Ard Mwampashi, Mandisa Singata-Madliki, Edna Arends, Elani Muller, Hadiza Galadanci, Zahida Qureshi, Fadhlun Alwy Al-Beity, Sue Fawcus, Neil Moran, George Gwako, Alfred Osoti, Ioannis Gallos, Arri Coomarasamy

**Affiliations:** 1 College of Medicine and Health, University of Birmingham, Birmingham, United Kingdom; 2 The UNDP/UNFPA/UNICEF/WHO/World Bank Special Programme of Research, Development and Research Training in Human Reproduction (HRP)/Department of Sexual, Reproductive, Maternal, Child, Adolescent Health and Ageing (LRH), World Health Organization, Geneva, Switzerland; 3 African Center of Excellence for Population Health and Policy, College of Health Sciences, Bayero University, Kano, Nigeria; 4 Department of Obstetrics and Gynecology, University of Nairobi, Nairobi, Kenya; 5 Department of Obstetrics and Gynecology, Muhimbili University of Health and Allied Sciences, Dar es Salaam, Tanzania; 6 Department of Obstetrics and Gynaecology, University of Cape Town, Cape Town, South Africa; 7 Effective Care Research Unit, University of the Witwatersrand, Johannesburg, South Africa; 8 Walter Sisulu University, Mthatha, South Africa; 9 KwaZulu-Natal Department of Health, Pietermaritzburg, South Africa; 10 Department of Obstetrics and Gynaecology, Nelson Mandela School of Medicine, University of KwaZulu-Natal, Durban, South Africa; 11 Nuffield Department of Women’s and Reproductive Health, John Radcliffe Hospital, University of Oxford, Oxford, United Kingdom

**Keywords:** postpartum haemorrhage (PPH), early detection, diagnostic methods, blood loss thresholds, maternal postpartum assessments

## Abstract

**Background:**

Postpartum haemorrhage (PPH) is the leading cause of maternal mortality, particularly in low- and- middle-income countries. The E-MOTIVE trial demonstrated a 60% reduction in severe PPH and related complications with the E-MOTIVE intervention compared to usual care. This nested observational study explored clinical care practices between the time of vaginal birth and the removal of the obstetric drape. Specifically, we assessed the frequency of postpartum maternal assessments, including blood pressure, pulse, uterine tone, vaginal blood flow, and cumulative blood loss assessment—unique to the intervention. We also evaluated diagnostic methods, and blood loss thresholds used for PPH, and how these practices may have contributed to differences in PPH diagnosis and outcomes between E-MOTIVE intervention hospitals and usual care hospitals.

**Methods:**

This prospective observational study, nested within the E-MOTIVE trial, involved passive, direct observations of healthcare workers providing postpartum care to women and managing PPH across 78 hospitals in Nigeria, Kenya, Tanzania, and South Africa. We conducted a descriptive analysis of the frequency and timing of postpartum maternal assessments, diagnostic methods and blood loss thresholds used to diagnose PPH, comparing clinical practices between E-MOTIVE care and usual care.

**Results:**

Between June and December 2022, the study included 2,578 women at E-MOTIVE care hospitals and 2,834 at usual care hospitals. At E-MOTIVE hospitals, 88% (2,272/2,578) of women received at least one postpartum maternal assessment, 71% (1,825/2,578) at least two, 57% (1,479/2,578) at least three, and 48% (1,234/2,578) four, with assessments conducted earlier and more frequently than at usual care hospitals. Objective blood loss quantification improved diagnosis, with the most common method and blood loss threshold at E-MOTIVE hospitals being ≥300 mL plus at least one abnormal clinical sign, used in 47% (140/295) of PPHs. Postpartum haemorrhage was diagnosed earlier and more frequently at E-MOTIVE hospitals: 76% (223/295) within 30 min, 97% (287/295) within 60 min, and 100% 295/295) within 90 min (median: 17 min; IQR 11–30), compared to 54% (119/219), 79% (173/219), 96% (210/219) respectively, and 100% within 134 min in usual care (median: 26 min; IQR 13–56).

**Discussion:**

Frequent and timely postpartum maternal assessments, along with objective blood loss thresholds of with at least one abnormal clinical sign and ≥500 mL may enhance early PPH diagnosis. The first 90 min postpartum has been identified as a critical window for early diagnosis, termed the “Golden 90 min for PPH diagnosis.” Incorporating these insights into clinical training and guidelines may support improved maternal outcomes related to PPH.

## 1 Introduction

Maternal mortality has been a major global public health concern for decades, with sub-Saharan Africa baring 70% of the burden (1). Postpartum haemorrhage (PPH), the leading cause of maternal mortality worldwide, accounts for 27% of maternal deaths (1). Despite advancements in obstetric care, challenges in early PPH diagnosis persist due to several factors. The 2014 World Health Organization (WHO) guidelines identified a major evidence gap regarding postpartum maternal assessments following vaginal birth. While periodic assessments during the immediate postpartum period (birth to 24 h) were recommended, there was a lack of studies exploring their impact on maternal mortality and morbidity. In 2022, WHO's recommendations on postpartum maternal assessments reverted to the 2014 guidelines due to the absence of new evidence (2–10). The universally used subjective visual estimation of blood loss is often inaccurate (11–14). Recent evidence demonstrates that blood loss thresholds below 500 mL, particularly ≥300 mL, when combined with abnormal haemodynamic signs such as tachycardia and hypotension, improves sensitivity and maintain acceptable specificity for predicting severe maternal morbidity and mortality (15). These findings provide a physiological basis for revisiting current maternal postpartum assessment guidelines.

The cluster-randomised E-MOTIVE trial (NCT04341662) evaluated a comprehensive clinical intervention for PPH diagnosis and treatment in women having vaginal birth (16). The intervention involved objective quantification of cumulative blood loss using a calibrated obstetric drape for early and accurate PPH diagnosis, and the use of a treatment bundle, with an implementation strategy. The treatment bundle, referred to as MOTIVE, comprised of multiple treatments to be administered concurrently or in parallel after a PPH diagnosis. MOTIVE stands for: uterine **M**assage, administration of **O**xytocics, **T**ranexamic acid, **IV** fluids, **E**xamination of the genital tract, and rapid escalation when needed. The control arm continued using visual estimation of blood loss and followed local guidelines for PPH treatment. The primary outcome was a composite of severe PPH (blood loss of ≥1,000 mL), or laparotomy due to bleeding, or maternal death caused by bleeding. The trial reported a 60% relative reduction in the primary outcome in the E-MOTIVE intervention arm compared with the control arm [1·6% vs. 4·3%; Risk Ratio: 0·40; 95% confidence interval (CI): 0·32 to 0·50; *P* < 0·001].

Since then, the WHO has updated its recommendations to include the E-MOTIVE intervention to support early diagnosis and timely management of PPH, aiming to prevent adverse maternal outcomes (17, 18). The updated guidance emphasises routine objective quantification of postpartum blood loss for PPH diagnosis, but does not specify any criterion for these assessments. Although these updated guidelines address various aspects, the specific clinical care processes related to early PPH diagnosis in the E-MOTIVE trial have not been fully explored between hospitals following E-MOTIVE care and those following usual care.

This nested observational study within the E-MOTIVE trial aimed to explore various aspects of clinical care within the initial hours after vaginal birth. Specifically, we evaluated how the frequency and timing of postpartum maternal assessments, the clinical signs they encompass, and the PPH diagnostic methods and blood loss thresholds used impact the timing and frequency of PPH diagnosis.

## 2 Methods

### 2.1 Study design

A multi-country cluster-randomised trial (registration number: NCT04341662) assessed the early diagnosis of PPH and a first response treatment bundle at 78 secondary and tertiary level hospitals in Nigeria, Kenya, Tanzania and South Africa (16). This trial had a 7-month baseline phase, then randomisation of the hospitals into either the intervention or control arm. To ensure balance between intervention and control facilities, a minimisation algorithm was used during randomisation—stratified by country—to account for factors such as birth volume, primary outcome rates, oxytocin quality, and the number of clusters per country. This was followed by a 2-month transition phase during which the hospitals allocated to the intervention attended training and embedded the intervention, and finally a 7-month post-randomisation phase, during which phase comparative effectiveness data were gathered.

Post randomisation, the intervention arm implemented “E-MOTIVE care”, consisting of early and accurate diagnosis of PPH by introducing a calibrated obstetric drape with graduated quantification lines, in millilitres (mL), to objectively assess cumulative vaginal blood loss in real-time. If PPH was diagnosed, a bundle of treatment, called “MOTIVE” was implemented. MOTIVE (in no set order) stood for uterine **M**assage, administration of **O**xytocics, **T**ranexamic acid, **IV** fluid, **E**xamination of the genital tract and rapid escalation when required (16). The control arm followed “usual care”, continued to use a non-calibrated obstetric drape, relied on subjective visual estimation of blood loss for PPH diagnosis, and adhered to local guidelines for PPH treatment.

At E-MOTIVE care hospitals, healthcare workers received training to follow specific postpartum maternal assessments and diagnostic criteria for PPH diagnosis. Maternal assessments consisted of assessing uterine tone, vaginal blood flow, and objective cumulative blood loss every 15 min for at least the first hour postpartum, and vital signs [blood pressure (BP) and pulse] at least once in the first hour postpartum.

At usual care hospitals, healthcare workers were advised to follow the WHO guidelines, recommending assessment of BP, pulse and uterine tone every 15 min for two hours (7) or to adhere to their local hospital guidelines or protocols. Local guidelines varied across countries and facilities, with differences in timing, frequency, and assessment components.

Both E-MOTIVE and usual care hospitals utilised clinical judgement and the WHO-defined PPH threshold (≥ 500 mL) (17) for PPH diagnosis. However, E-MOTIVE care hospitals employed objective quantification of cumulative blood loss using a graduated calibrated drape, while usual care hospitals relied on subjective visual estimation using a non-calibrated obstetric drape. Additionally, E-MOTIVE care hospitals had the option of applying a blood loss threshold of ≥300 mL combined with at least one abnormal clinical sign or additional clinical concern such as anaemia (Supplementary Appendix 1, Table 1 and 2). The predefined clinical signs indicating abnormality were as follows: pulse >100 beats per minute (bpm) or an increase of ≥20 bpm from baseline; systolic BP <100 mmHg or a drop of ≥20 mmHg from baseline; soft uterine tone; heavy vaginal blood flow, large clots, or a constant trickle. Thus, the three criteria for initiating the treatment bundle in the E-MOTIVE hospitals were: (1) clinical judgement that PPH treatment was needed (regardless of measured blood loss), (2) measured blood loss ≥500 mL (regardless of other clinical signs), and (3) measured blood loss of ≥300 mL plus at least one abnormal clinical sign or clinical concern. Details of the E-MOTIVE trial are published elsewhere (16).

Nested within the E-MOTIVE trial, we conducted an observational study that used direct passive prospective observations of healthcare workers providing clinical care to women during vaginal birth, providing postpartum care to women and managing PPH (if occurred) at 78 hospitals participating in the E-MOTIVE trial between June and December 2022. Observations were conducted from the time of vaginal birth, until the removal of the obstetric drape.

The E-MOTIVE trial was approved by the relevant ethics and regulatory review committees of each country (Supplementary Appendix 2). Individual-level consent from women for observations was not obtained, because they were not the target of the E-MOTIVE intervention, they were not interacted with for data collection, and no identifiable information was recorded. The study adhered to the principles of the Declaration of Helsinki, CIOMS International Ethical Guidelines, and the Ottawa Statement for the Ethical Design and Conduct of Cluster Randomised Trials.

Information regarding the E-MOTIVE trial (16), as well as further analyses based on this observational study (19–21), are available in other publications.

### 2.2 Nested study procedures

Observations of healthcare workers (midwives, nurse-midwives, nurses) providing clinical care to women were conducted consecutively during the day, overnight, weekdays, and weekends, over a one-to-two-week period to reduce selection bias and capture instances of PPH. There were no exclusion criteria imposed on this nested study. However, if a woman was excluded from the primary E-MOTIVE trial they were excluded from the nested study. Observers transitioned from a passive to an active role on some occasions if PPH became life-threatening, necessitating clinical intervention for the woman's safety.

Implementation midwives and research midwives employed by the trial conducted the observations. All 148 observers attended one of the 17 standardised two-hour training session conducted via Zoom by the same trainer (K-MM). These sessions included an overview of the observation rationale, an explanation of the importance of passive observation and how to conduct them, a detailed review of each question in the structured guide, and a dedicated question and answer segment. All sessions were recorded and shared with participants to ensure accessibility in case of technical issues.

Following the session, observers completed practice (dummy) entries in a test REDCap project. This allowed them to build confidence and familiarity with the tool, improve accuracy in data entry, and surface any potential challenges. All practice entries were reviewed, with follow-up guidance provided as needed. Observers were only granted access to the live REDCap database once their competency and understanding were confirmed.

Training was delivered in a staggered manner, aligned with the E-MOTIVE trial's randomisation schedule, and conducted one to three weeks prior to the commencement of data collection. The timing of each session varied according to logistical constraints and the availability of both the trainer and site observers.

To ensure consistency across sites, all observers used a paper version of the structured observation guide to record findings in real time. These were then entered into the REDCap system as soon as possible following each observation, minimising recall bias and supporting standardised data collection. To further ensure data accuracy and reliability, a structured oversight process was implemented. All observational entries were reviewed daily by the data manager (K-MM), a Clinical Midwife Specialist with over two decades of combined clinical and research experience. Reviews were conducted seven days a week throughout the six-month data collection period, allowing for timely identification and resolution of any omissions, inconsistencies, or invalid entries. Queries were raised within 24 h of data entry to maximise observer recall and facilitate access to relevant clinical records. Any unresolved queries exceeding 48 h were followed up directly, with observers receiving targeted support to ensure clarity and consistency. This continuous and clinically informed review process provided a robust framework for maintaining data integrity and addressing potential errors in real time.

Two structured observation guides were employed: one for hospitals implementing E-MOTIVE care and another for usual care (Supplementary Appendix 3). The primary difference was the inclusion of care related to the calibrated drape at E-MOTIVE care hospitals. Both guides encompass patient characteristics, time of birth, drape funnel opening and removal, active management of third stage, and postpartum maternal assessments, including clinical signs documented on the blood loss monitoring chart specifically designed for the E-MOTIVE trial (Supplementary Appendix 3), and for the usual care hospitals on the postnatal monitoring chart routinely used. For PPHs diagnosed, time of PPH diagnosis, PPH diagnostic methods and thresholds, treatments administered, and escalation of care procedures, were documented with start and stop timestamps. Additionally, medication doses and the healthcare worker cadre responsible for each aspect of care were recorded. To avoid replication of data entry, the following data was obtained from the primary E-MOTIVE trial database from those observed: maternal age, gestational age at birth, type of pregnancy, number of previous births ≥ 24 weeks, previous caesarean section, PPH in a previous pregnancy, episiotomy, perineal tear, laparotomy for bleeding, blood transfusion, intensive care admission, and final blood loss quantification as per the source verified drape weight.

### 2.3 Outcomes

**Figure 1:**
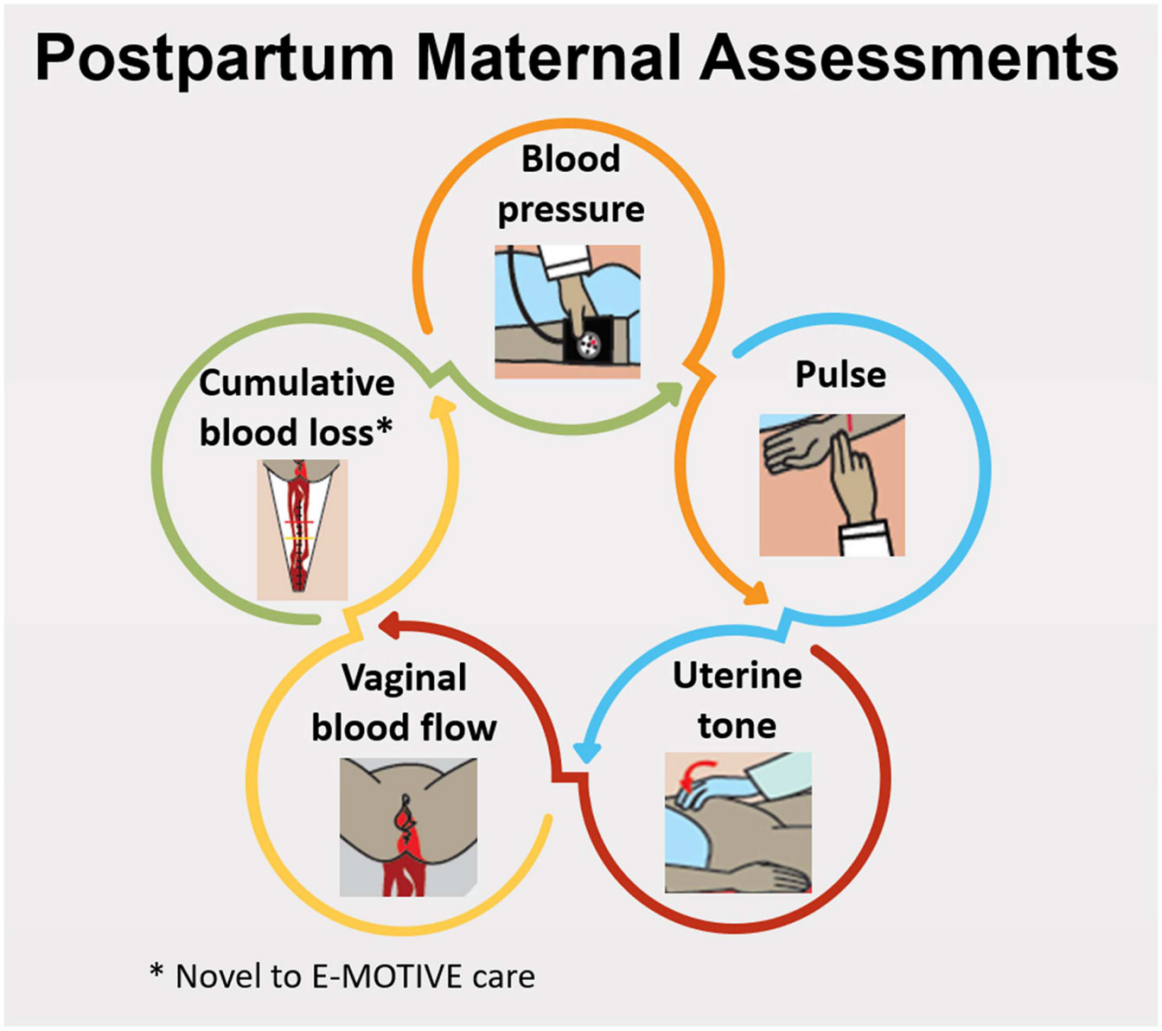
Postpartum maternal assessments and clinical signs for postpartum haemorrhage diagnosis.

Postpartum maternal assessments conducted by healthcare workers correspond to instances where a healthcare worker assessed a woman's health status, with a maximum of four assessments, generally within the first hour after childbirth, encompassing the examination of clinical signs (Figure 1).

The outcome measures reported in this analysis are:

1. Clinical care practices from a woman's vaginal birth until the obstetric drape was removed or PPH was diagnosed: (i) Frequency and timing of each maternal assessment, the clinical signs within each assessment, and only in E-MOTIVE care hospitals, the assessment of objective cumulative blood loss using the calibrated drape; (ii) Frequency and timing of PPH diagnosis; and (iii) Frequency and timing of each PPH diagnostic method and blood loss threshold.
2. Adherence to the postpartum maternal assessment criteria, which involved the following within the initial 65 min postpartum: assessment of uterine tone, vaginal blood flow, and objective cumulative blood loss every 15 min, and assessment of vital signs (BP and pulse) at least once in the first hour after childbirth.

### 2.4 Analysis

A subgroup representing approximately 5% of vaginal births from the implementation phase of the E-MOTIVE trial (99,659 women) was estimated during the predetermined observation period. The necessary timeframe for achieving the estimated number of observations predicted was based on historical data collected from each hospital during the E-MOTIVE trial. This pragmatic approach was chosen to fit within the trial's timeline constraints*.*

Descriptive analysis was undertaken using STATA 18, stratified by E-MOTIVE care and usual care hospitals. Continuous measures were reported as medians and interquartile ranges (IQR) due to skewness. Categorical data were reported as frequencies and percentages. All analyses were conducted using complete case analysis, as it was expected that there would be minimal missing data across the variables. The clinical care pathway analysed was from vaginal birth and continued until a healthcare worker diagnosed PPH, or for women without a PPH diagnosed, the analysis extended until the obstetric drape was removed.

## 3 Results

**Figure 2:**
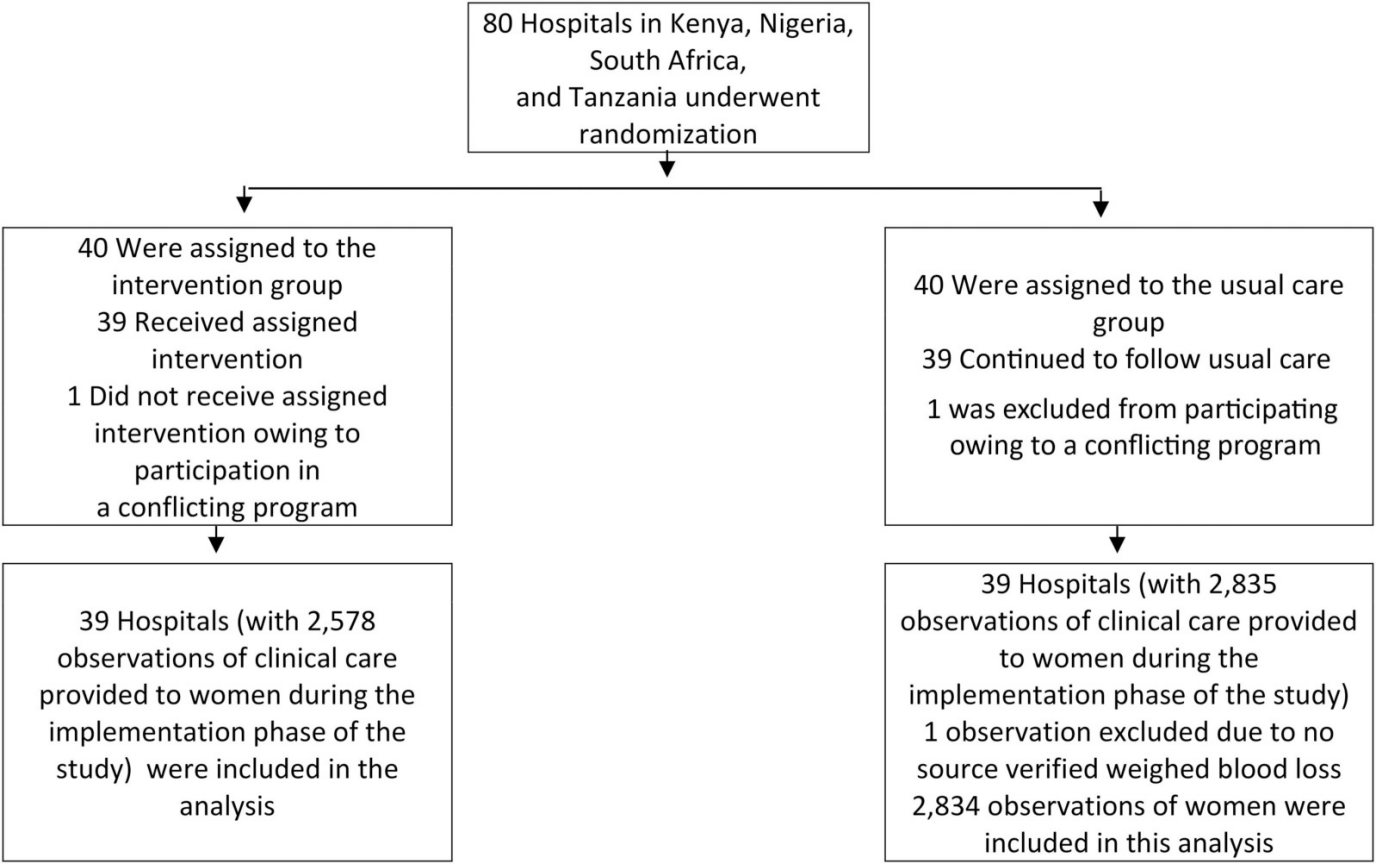
Consort diagram: observations.

Clinical care delivered by healthcare workers for a total of 5,413 women within the first few hours postpartum were directly observed (2,578 in E-MOTIVE care; 2,835 in usual care) at the 78 participating hospitals (39 hospitals assigned to E-MOTIVE care; 39 hospitals following usual care), across Nigeria, Kenya, Tanzania, and South Africa (Figure 2). Only one observation was excluded because source data verification of the final objective blood loss quantification after the drape was removed and weighed was missing, leaving 5,412 observations included in this analysis. In total, 514 women had a PPH (295 in E-MOTIVE care; 219 in usual care), defined as a woman who had a PPH clinically diagnosed by a healthcare worker, regardless of the final objective blood loss quantification after the drape was removed and weighed.

Baseline characteristics of the enrolled women were largely similar across the E-MOTIVE and usual care hospitals, with some notable differences (Table 1). All baseline characteristics are listed in Supplementary Appendix 1, Table 3. Haemoglobin was tested less often at E-MOTIVE care hospitals (56%, 1,433/2,578), compared with usual care hospitals (67%, 1,904/2,834). Augmentation of labour was less frequent at E-MOTIVE care hospitals (5%, 117/2,578) compared with usual care hospitals (16%, 444/2,834). Placental checks as part of active management of third stage were also less frequent at E-MOTIVE care hospitals (68%, 1,761/2,578) than at usual care hospitals (92%, 2,599/2,834).

**Table 1:** Baseline characteristics.

Baseline characteristics	E-MOTIVE care *N* = 2,578	Usual care *N* = 2,834
Pregnancy information		
Maternal age, median[IQR]	25 [21–30]	25 [22–30]
Gestational age at birth, median[IQR]	38 [37–40]	38 [37–39]
Previous caesarean section, [*n*, (%)]	64 (2·48)	73 (2·58)
Previous postpartum haemorrhage, [*n*, (%)]	27 (1·05)	24 (0·85)
Health conditions		
Body mass index, median[IQR]	24·81 [23·01–27·73]	25·2 [22·77–28·4]
Hypertension, [*n*, (%)]	48 (1·86)	53 (1·87)
Diabetes, [*n*, (%)]	5 (0·19)	4 (0·14)
Malaria, [*n*, (%)]	11 (0·43)	160 (5·65)
Pregnancy, labour, birth risk factors		
Frequency of haemoglobin testing in pregnancy, [*n*, (%)]	1,433 (55·59)	1,904 (67·18)
Haemoglobin levels, median[IQR]	101 [94–106]	100 [95–104]
Anaemic^a^	574 (22.27)	629 (22.19)
Placental abruption, [*n*, (%)]	9 (0·35)	14 (0·49)
Pregnancy induced hypertension, [*n*, (%)]	73 (2·83)	72 (2·54)
Pre-eclampsia, [*n*, (%)]	54 (2·09)	45 (1·59)
Eclampsia, [*n*, (%)]	15 (0·58)	10 (0·35)
Antepartum/intrapartum haemorrhage, [*n*, (%)]	13 (0·5)	44 (1·55)
Pushing > 60 min, [*n*, (%)]	43 (1·67)	132 (4·66)
Induction of labour, [*n*, (%)]	112 (4·34)	184 (6·49)
Augmentation of labour, [*n*, (%)]	117 (4·54)	444 (15·67)
Compound presentation, [*n*, (%)]	4 (0·16)	71 (2·51)
Breech presentation, [*n*, (%)]	26 (1·01)	40 (1·41)
Episiotomy, [*n*, (%)]	341 (13·23)	432 (15·24)
Vaginal/perineal tear, [*n*, (%)]	551 (21·37)	683 (24·1)
Shoulder dystocia, [*n*, (%)]	9 (0·35)	8 (0·28)
Study procedures		
Time from vaginal birth to drape funnel opening (minutes), Median [IQR]	2 [1–3]	2 [1–3]
AMTSL: medicines administered		
Oxytocin, [*n*, (%)]	2,535 (98·33)	2,829 (99·82)
Misoprostol, [*n*, (%)]	817 (31·69)	850 (29·99)
Ergometrine, [*n*, (%)]	1 (0·04)	6 (0·21)
Carbetocin, [*n*, (%)]	42 (1·63)	9 (0·32)
AMTSL: management of the placenta		
Controlled cord traction performed, [*n*, (%)]	2,555 (99·11)	2,814 (99·29)
Manual removal of placenta performed, [*n*, (%)]	23 (0·89)	20 (0·71)
Placenta checked, [*n*, (%)]	1,761 (68·31)	2,599 (91·71)

SD, standard deviation; IQR, interquartile range; n, number; %, percentage; AMTSL, active management of third stage of labour.

a Anaemic < 110 grams per litre^1^.

### 3.1 Postpartum maternal assessments conducted

**Figure 3:**
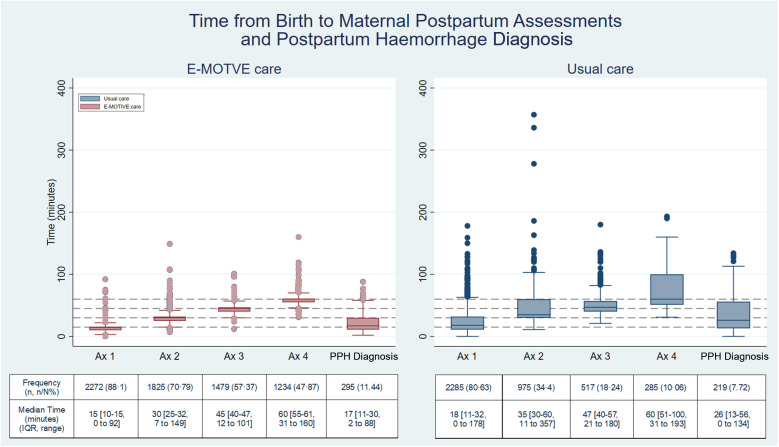
Time from birth to postpartum maternal assessments and postpartum haemorrhage diagnosis. E-MOTIVE care *N* = 2,578; Usual care *N* = 2,834; Ax = postpartum maternal assessment; PPH, postpartum haemorrhage; Percentage(%) =number**(n)**/total number**(N)**; IQR, interquartile range.

At E-MOTIVE care hospitals, 88% (2,272/2,578) of women had at least one assessment where a healthcare worker assessed their health status, 71% (1,825/2,578) had at least two assessments, 57% (1,479/2,578) had at least three assessments, and 48% (1,234/2,578) had four assessments. A maximum of four assessments per woman were conducted and observed. The median times for each of these assessments were 15 min (IQR 10–15), 30 min (IQR 25–32), 45 min (IQR 40–47), and 60 min (IQR 55–61) respectively (Figure 3). The proportion of women assessed in E-MOTIVE hospitals was consistently higher for any given maternal assessment and performed earlier than in usual care hospitals (Figure 3). When a maternal assessment was conducted, there were no substantial differences in the frequencies of each clinical sign evaluated at each assessment between the E-MOTIVE and usual care hospitals (Supplementary Appendix 1, Table 4).

Finally, in the E-MOTIVE care hospitals, 12% (310/2,578) of women never had an assessment (no clinical sign was assessed) within the initial 65 min postpartum, compared with 22% of (622/2,834) of women in the usual care hospitals. Full adherence to the E-MOTIVE postpartum maternal assessment guidance criteria within 65 min postpartum was achieved in 20% (518/2,578) of women in E-MOTIVE care, compared with 2% (53/2,834) in usual care (Supplementary Appendix 1, Table 5).

### 3.2 Calibrated drape use

Regarding PPH diagnosis at the E-MOTIVE care hospitals, there were no substantial differences in the frequency of the calibrated drape assessments compared with all other clinical signs during each assessment (Supplementary Appendix 1, Table 5). The calibrated drape was assessed during the first assessment between 10 and 20 min postpartum in 62% (1,597/2,578) of women, the second assessment between 25 and 35 min in 46% (1,180/2,578) of women, the third assessment between 40 and 50 min in 36% (936/2,578) of women and the fourth assessment between 55 and 65 min in 31% (797/2,578) of women. Full adherence to the E-MOTIVE postpartum maternal assessment guidance criteria within 65 min postpartum for the calibrated drape assessment was achieved in 28% (717/2,578) of women.

### 3.3 Timing from birth to postpartum haemorrhage diagnosis

The median time from birth to clinically diagnosed PPH were 17 min (IQR 11–30) in E-MOTIVE care, compared with 26 min (IQR 13–56) in usual care. Postpartum haemorrhages were diagnosed quicker and within a narrower time frame in E-MOTIVE care compared with usual care. In E-MOTIVE hospitals, 76% (223/295) of women with a PPH were diagnosed within the first 30 min postpartum, 22% (64/295) between 31 and 60 min, and 3% (8/295) between 61 and 90 min. In usual care 54% (119/219) were diagnosed within the first 30 min postpartum, 25% (54/219) between 31 and 60 min, 17% (37/219) between 61 and 90 min and the remaining 4% (9/219) after 90 min (Figure 3).

### 3.4 Diagnostic methods used

**Figure 4:**
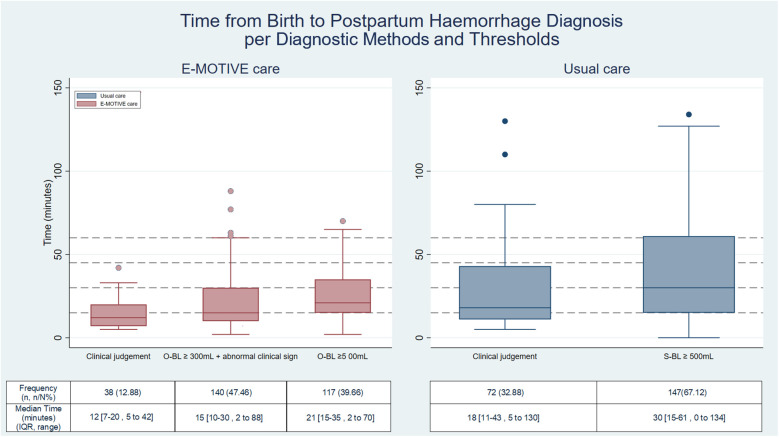
Time to postpartum haemorrhage per diagnosis method and threshold*.* Clinically diagnosed postpartum haemorrhages: E-MOTIVE care = 295, Usual care = 219; S-BL, subjective blood loss; O-BL, objective blood loss; Percentage(%) =number**(n)**/total number**(N)**; IQR, interquartile range.

In E-MOTIVE care hospitals, the breakdown of PPH diagnostic methods and blood loss thresholds is as follows: 47% (140/295) of women experiencing PPH were identified through the objective blood loss threshold of ≥300 mL combined with at least one abnormal clinical sign, 40% (117/295) were identified using the objective threshold of blood loss ≥500 mL, and 13% (38/295) were identified through clinical judgement. In usual care, 67% (147/219) were identified through the subjective blood loss threshold of ≥500 mL and 33% (72/219) were identified by clinical judgement (Figure 4).

## 4 Discussion

This nested study within the E-MOTIVE trial demonstrates that the E-MOTIVE care intervention resulted in more timely and frequent postpartum maternal assessments compared with usual care. Across all four assessments within the first 65 min postpartum, women attended to in E-MOTIVE care hospitals consistently received more frequent initial and subsequent evaluations of their health status. Although both care approaches showed a decreasing trend in the frequency of assessments and clinical signs over time, this decline was less steep in E-MOTIVE care. Notably, almost 50% of women in E-MOTIVE care received all four assessments, compared with only 10% in usual care.

Our findings show that E-MOTIVE care hospitals diagnosed 76% of PPHs within the first 30 min postpartum, compared to 54% in usual care hospitals. This highlights the critical nature of early diagnosis through timely and frequent maternal assessments, especially as the last PPH was diagnosed at 88 min postpartum in E-MOTIVE care, compared to 134 min in usual care. This earlier diagnosis is likely attributable to the structured postpartum maternal assessments incorporated into training and supportive documentation, along with the use of calibrated drapes, which enabled healthcare workers to identify abnormal bleeding more rapidly and consistently. This introduces the concept of the “Golden 90 min for PPH diagnosis”, a critical window to diagnose PPH. Clinically, the first hour postpartum is when massive PPH (≥1,500 mL) usually occurs (22) and most PPH-related deaths occur within two to three hours postpartum (6, 23, 24). During this time, uterine atony, the leading cause of PPH, can develop and worsen quickly if not identified and managed promptly. Early diagnosis is crucial, as delays in recognition and treatment can increase the risk of severe bleeding, shock, and maternal mortality. Frequent monitoring of all clinical signs during this period can lead to prompt diagnosis and management of PPH, helping to mitigate PPH-related adverse maternal outcomes. Moreover, 87% of those PPHs were diagnosed through monitoring the calibrated drape, whereas in usual care hospitals, diagnosis was achieved through visual estimation of blood loss (67%) or clinical judgement (33%). It is worth noting that nearly half of the PPHs in E-MOTIVE care hospitals were identified using the calibrated drape, which incorporated the ≥300 mL blood loss threshold combined with at least one abnormal clinical sign.

The consistent advantages of E-MOTIVE care in early PPH diagnosis could be due to several key mechanisms. Women in E-MOTIVE care hospitals received more frequent and timely postpartum assessments compared to those in usual care, without significant differences in the clinical signs evaluated during these assessments. The use of a calibrated drape for objective blood loss quantification facilitated timely identification of PPH, contrasting with the subjective visual estimation methods commonly employed in usual care. These mechanisms worked synergistically to reduce delays in recognition, allowing for faster clinical decision-making and intervention.

Current research on postpartum maternal assessments largely relies on small observational and retrospective studies, often hindered by incomplete or inconsistent medical records (25–27). The lack of data on postpartum maternal assessments after vaginal birth impedes guideline development (2–10). Our findings at usual care hospitals align with studies from Burkina Faso and Côte d'Ivoire, which similarly highlight inadequate postpartum maternal assessments during the critical initial hours postpartum (28). These findings highlight an urgent need to strengthen clinical practices and address human resource constraints in LMICs (29). Implementing task shifting to other trained cadres beyond midwives or nurse-midwives, may enhance timely postpartum maternal assessments and facilitate early identification of abnormal findings. Neglecting these assessments risks missed or delayed PPH diagnosis, significantly increasing the risk of severe maternal complications (28).

In low-resource settings, early PPH diagnosis has traditionally relied on subjective visual estimation of blood loss, which is widely used but shown to be highly inaccurate (11–14). The use of calibrated drapes alone has been introduced as an objective tool, but a multi-country evaluation by Zhang et al., demonstrated that the drape alone did not significantly reduce severe PPH (≥1,000 mL) (30). In contrast, the E-MOTIVE care bundle is an advancement over these traditional methods. Unlike visual estimation or the calibrated drape used in isolation, E-MOTIVE combines objective measurement of blood loss with regular and timely, structured postpartum maternal assessments, defined thresholds for abnormal clinical signs, diagnostic methods, prompt protocol-driven actions. These key mechanisms supported earlier PPH diagnosis and may have contributed to reduced adverse outcomes for women during the E-MOTIVE trial, including a lower risk of blood loss ≥1,000 mL.

The recent FIGO, ICM and WHO Consolidated Guidelines on the Prevention and Treatment of Postpartum Haemorrhage has redefined PPH as ≥300 mL of blood loss accompanied by at least one abnormal clinical sign, reflecting a global shift towards earlier diagnosis and intervention (18). This definition aligns closely with the diagnostic threshold used in the E-MOTIVE trial, supporting its validity and potential for broader adoption. By lowering the diagnosis threshold and incorporating clinical signs, the updated definition affirms the importance of timely, objective quantification of blood loss combined with clinical assessment to prevent progression to severe PPH.

Effective postpartum monitoring extends beyond routine assessments; it involves leveraging gathered information for real-time clinical decision-making to facilitate prompt treatment (28, 31, 32). Our study illustrates these principles, showing that E-MOTIVE training led to more consistent postpartum maternal assessments for early PPH diagnosis, regarding both frequency and timing. The training emphasised applying E-MOTIVE's PPH diagnosis criteria, which include abnormal clinical sign parameters, objective blood loss quantification, and diagnostic methods and blood loss thresholds. Consequently, healthcare workers improved their efficiency in diagnosing PPHs by combining maternal assessments with the new criteria of ≥300 mL blood loss combined with at least one abnormal clinical sign, a safety net threshold of ≥500 mL, and clinical judgement.

The strengths of our study include its integration within a large, multi-country, cluster-randomised trial, which allowed for the adjustment of confounding factors prior to observations. To mitigate potential “Hawthorne effects”, the implementation and research midwives conducting the observations had pre-existing rapport with hospital staff, aiming to minimise disruptions and changes in staff behaviour (33). Previous studies have indicated that healthcare workers often become less aware of being observed over time, focusing more on their tasks (34, 35). However, we acknowledge that a risk of observer bias remained. Despite standardised training and efforts to reduce this risk, such as using staff employed by the primary study and the use of structured observation tools, observer interpretation may still have influenced the way clinical care was recorded, potentially affecting the consistency or objectivity of the data. While formal inter-observer reliability assessments (e.g., double-coded observations) were not feasible within the study's operational context, observer consistency was supported through training, supervision, and use of structured tools across hospitals. This study focused on describing adherence and implementation patterns of postpartum maternal assessments between E-MOTIVE care and usual care hospitals, however further work could explore predictors of adherence and early PPH diagnosis using multivariable analysis. Examining factors such as country, facility level, and staff cadre could provide valuable insights into contextual determinants of implementation fidelity.

E-MOTIVE care, including frequent maternal postpartum assessments, requires appropriate implementation support to ensure consistent delivery across diverse health system contexts. Findings from the E-MOTIVE process evaluation (19) highlight several implementation challenges that influenced adherence, including staffing shortages, time constraints, and competing clinical demands. While training was generally well received, gaps in ongoing supervision and reinforcement of protocol components were noted, particularly in high-volume or understaffed facilities. These factors affected the consistency of postpartum assessments and highlight the importance of embedding supportive strategies, such as refresher training, workflow integration, and leadership engagement, into routine practice. Addressing these challenges will be critical to ensuring fidelity, feasibility, and long-term sustainability of E-MOTIVE care in real-world health systems.

A separate cost-effectiveness analysis conducted alongside the E-MOTIVE trial found that early diagnosis of PPH using a calibrated drape combined with bundled WHO-recommended treatment was cost-effective compared to usual care (36). The findings suggest that while cost-effective at scale, implementation costs can be absorbed within existing health systems, making E-MOTIVE a feasible and efficient use of limited healthcare resources.

However, scaling up the E-MOTIVE care intervention could face many logistical, financial and cultural barriers. Logistically, getting the necessary supplies (including uterotonics and calibrated drapes) available consistently, training of providers, and integrating the intervention into existing workflows could be challenging in under-resourced areas. Financial constraints (limited national health budgets and competing priorities) may limit investment in procurement, training and supervision. Different beliefs about childbirth practices and reluctance to adopt new protocols could also affect acceptance and adherence among providers and communities. Context-specific implementation strategies, stakeholder engagement and policy support will be essential to sustain the impact of the intervention.

In summary, the E-MOTIVE trial demonstrated a significant improvement in early PPH diagnosis. This nested study identified several key mechanisms used in the trial that facilitated early diagnosis, which should be integrated into scaling up E-MOTIVE care. The combination of both frequent and timely assessments of all clinical signs, together with the diagnostic method of the objective measurement of ≥300 mL blood loss threshold combined with at least one abnormal clinical sign contributed to the prompt PPH diagnosis seen in E-MOTIVE care compared to usual care.

Future training should emphasise comprehensive postpartum maternal assessments every 15 min for the first hour and once at 90 min postpartum. Specific parameters for identifying abnormal clinical signs should guide the integration of clinical information with objective blood loss quantification, incorporate a blood loss threshold of ≥300 mL combined with at least one abnormal clinical sign, a safety net threshold of ≥500 mL, and clinical judgement. Recognising the first 90 min postpartum as the “Golden 90 min for PPH diagnosis” is crucial for timely identification. These criteria and clinical practices should be clearly translated into clinical guidelines and protocols to ensure consistent implementation across settings. By adopting these practices, healthcare workers can enhance early PPH diagnosis, leading to prompt treatment and improved maternal health outcomes. While long-term evidence is not yet available given the recent implementation of E-MOTIVE care, these improvements may, over time, contribute to substantial reductions in severe morbidity and maternal mortality associated with postpartum haemorrhage.

## Data Availability

The raw data supporting the conclusions of this article will be made available by the authors, without undue reservation.

## Footnotes

^1^World Health Organization. Haemoglobin concentrations for the diagnosis of anaemia and assessment of severity. Vitamin and Mineral Nutrition Information System. Geneva: World Health Organization. (2011).
