# Supplementary material for: Clinical care processes for early postpartum haemorrhage diagnosis: a nested observational study within the E-MOTIVE trial
Source: Front Glob Womens Health. 2025 Nov 24;6:1527261. doi: 10.3389/fgwh.2025.1527261 (PMC12682830; doi:10.3389/fgwh.2025.1527261)
Supplement: Supplementary file 1 [file Datasheet1.pdf]

Appendix 1: Tables

Table 1: Definitions of clinical diagnostic methods thresholds: E-MOTIVE care and E-usual care

Clinical postpartum haemorrhage diagnostic methods and thresholds	Definitions	
	Hospitals implementing E-MOTIVE care	Hospitals following usual care
Clinical judgement	"A reflective and reasoning process that draws upon all available data is informed by an extensive knowledge base and results in the formation of a clinical conclusion" (1, pp. 3337)	"A reflective and reasoning process that draws upon all available data is informed by an extensive knowledge base and results in the formation of a clinical conclusion" (1, pp. 3337)
≥ 500 mL blood loss	Objective quantification of cumulative blood loss using calibration lines on an obstetric drape funnel*	Subjective visual estimate of cumulative blood loss
≥ 300 mL blood loss + abnormal clinical sign(s)*	The real-time objective quantification of cumulative blood loss was achieved through using the calibration lines on the obstetric drape. This, combined with the criteria for abnormal clinical signs based on postpartum maternal assessments during the E-MOTIVE trial* (Table: 2)	n/a

CAPTION: *Novel to E-MOTIVE care hospitals

Table 2: Abnormal clinical sign parameters applied to E-MOTIVE care hospitals

Clinical sign checked	Abnormal parameters of clinical signs
Pulse	Abnormal if > 100 bpm OR an increase of ≥ 20 bpm from baseline (bpm = beats per minute)
Blood Pressure (BP)	Abnormal if systolic < 100 mmHg OR a systolic decrease of 20 mmHg from baseline
Uterine Tone	Abnormal if Soft
Vaginal blood flow	Abnormal if Heavy flow of PV blood loss/clinical PPH diagnosis<br>(PV = Per Vaginal)<br>OR Large blood clots expelled OR Constant trickle
Calibrated measurement line*	Abnormal if between 300 mL – 499 mL with at least one abnormal clinical sign OR<br>Abnormal if ≥ 500 mL

CAPTION: *Novel to E-MOTIVE care hospitals

[1] Connor, J., Flenady, T., Massey, D., Dwyer, T. Clinical judgement in nursing - An evolutionary concept analysis. J Clin Nurs. (2022). 32(13-14): 3328–3340. doi: 10.1111/jocn.16469.

Table 3: All baseline characteristics

	E-MOTIVE care	Usual care
	N=2,578	N=2,834
**Pregnancy information**		
Maternal age, median[IQR]	25 [21-30]	25 [22-30]
Gestational age at birth, median[IQR]	38 [37-40]	38 [37-39]
Previous caesarean section, (n, [%])	64 (2.48)	73 (2.58)
Previous postpartum haemorrhage, (n, [%])	27 (1.05)	24 (0.85)
**Health conditions**		
Body mass index, median[IQR]	24.81 [23.01-27.73]	25.2 [22.77-28.4]
Hypertension, (n, [%])	48 (1.86)	53 (1.87)
Diabetes, (n, [%])	5 (0.19)	4 (0.14)
Coagulation disorder, (n, [%])	0 (0)	1 (0.04)
Heart disease, (n, [%])	2 (0.08)	1 (0.04)
Kidney disease, (n, [%])	1 (0.04)	1 (0.04)
Malaria, (n, [%])	11 (0.43)	160 (5.65)
Uterine fibroids, (n, [%])	1 (0.04)	8 (0.28)
**Pregnancy, labour, birth risk factors**		
Frequency of haemoglobin testing in pregnancy, (n, [%])	1433 (55·59)	1904 (67·18)
Haemoglobin, median[IQR]	101 [94-106]	100 [95-104]
Anaemic**	574 (22.27)	629 (22.19)
Placenta previa or low lying, accreta, increta or percreta, (n, [%])	5 (0.19)	9 (0.32)
Placental abruption, (n, [%])	9 (0.35)	14 (0.49)
Chorioamnionitis, (n, [%])	3 (0.12)	10 (0.35)
Pregnancy induced hypertension, (n, [%])	73 (2.83)	72 (2.54)
Pre-eclampsia, (n, [%])	54 (2.09)	45 (1.59)
Eclampsia, (n, [%])	15 (0.58)	10 (0.35)
Antepartum/intrapartum haemorrhage, (n, [%])	13 (0.5)	44 (1.55)
Febrile in labour (temperature >38°C), (n, [%])	5 (0.19)	25 (0.88)
Pushing > 60 minutes, (n, [%])	43 (1.67)	132 (4.66)
Induction of labour, (n, [%])	112 (4.34)	184 (6.49)
Augmentation of labour, (n, [%])	117 (4.54)	444 (15.67)
Compound presentation, (n, [%])	4 (0.16)	71 (2.51)
Breech presentation, (n, [%])	26 (1.01)	40 (1.41)
Malpresentation or malposition, (n, [%])	9 (0.35)	12 (0.42)
Episiotomy, (n, [%])	341 (13.23)	432 (15.24)
Vaginal/perineal tear, (n, [%])	551 (21.37)	683 (24.1)
Shoulder dystocia, (n, [%])	9 (0.35)	8 (0.28)
**Baby 1 - mode of birth**		
Spontaneous vaginal, (n, [%])	2566 (99.53)	2824 (99.65)
Forceps, (n, [%])	3 (0.12)	1 (0.04)
Ventouse, (n, [%])	9 (0.35)	9 (0.32)
obstructed labour, (n, [%])	1 (0.04)	0 (0)
Semi-conscious, (n, [%])	0 (0)	2 (0.07)

Obstetric drape funnel		
Time from vaginal birth to drape funnel opening (minutes), Median (IQR, range)	2 [1-3 , 0 to 14]	2 [1-3 , 0 to 17]
AMTSL: Medicines administered		
Oxytocin (n, [%])	2535 (98.33)	2829 (99.82)
Misoprostol (n, [%])	817 (31.69)	850 (29.99)
Ergometrine (n, [%])	1 (0.04)	6 (0.21)
Carbetocin (n, [%])	42 (1.63)	9 (0.32)
AMTSL: Management of the placenta		
Controlled cord traction performed, (n, [%])	2555 (99.11)	2814 (99.29)
Manual removal of placenta performed, (n, [%])	23 (0.89)	20 (0.71)
Placenta checked, (n, [%])	1761 (68.31)	2599 (91.71)

CAPTION: IQR=interquartile range; n=number; %=Percentage; AMTSL= Active management of third stage of labour; ** Anaemic < 110 grams per litre (2)

Table 4: Clinical signs assessed at each postpartum maternal assessment for postpartum haemorrhage diagnosis

Type of clinical signs assessed	Sets of clinical assessments	E-MOTIVE care N=2,578 (n, [%])	Usual care N=2,834 (n, [%])
Blood pressure	0 Ax	49 (2)	335 (12)
	1st Ax	2223 (86)	1950 (69)
	2nd Ax	1681 (65)	868 (31)
	3rd Ax	1337 (52)	490 (17)
	4th Ax	1142 (44)	280 (10)
Pulse	0 Ax	57 (2)	505 (18)
	1st Ax	2215 (86)	1780 (63)
	2nd Ax	1675 (65)	855 (30)
	3rd Ax	1336 (52)	487 (17)
	4th Ax	1137 (44)	278 (10)
Uterine tone	0 Ax	19 (1)	221 (8)
	1st Ax	2253 (87)	2064 (73)
	2nd Ax	1795 (70)	867 (31)
	3rd Ax	1439 (56)	499 (18)
	4th Ax	1202 (47)	279 (10)
Vaginal blood flow	0 Ax	22 (1)	181 (6)
	1st Ax	2250 (87)	2104 (74)
	2nd Ax	1790 (69)	879 (31)
	3rd Ax	1441 (56)	459 (16)
	4th Ax	1214 (47)	238 (8)

CAPTION: Ax= assessment; n=number; %=Percentage

[2] World Health Organization. Haemoglobin concentrations for the diagnosis of anaemia and assessment of severity. Vitamin and Mineral Nutrition Information System. Geneva: World Health Organization.(2011).

Table 5: Adherence criterion (birth to 65 minutes postpartum)

Time increments of each assessment (minutes)	E-MOTIVE care N=2,578 (n, n/N %)						Usual care N=2,834 (n, n/N %)				
	Blood pressure	Pulse	Uterine tone	Vaginal blood flow	4 clinical signs ~ per time increment	Calibration line †	Blood pressure	Pulse	Uterine tone	Vaginal blood flow	4 clinical signs ~ per time increment
1st - 10-20	1617 (62.72)	1612 (62.53)	1624 (62.99)	1618 (62.76)	1590 (61.68)	1597 (61.95)	732 (25.83)	685 (24.17)	833 (29.39)	824 (29.08)	640 (22.58)
2nd - 25-35	1138 (44.14)	1133 (43.95)	1184 (45.93)	1179 (45.73)	1106 (42.9)	1180 (45.77)	349 (12.31)	347 (12.24)	355 (12.53)	356 (12.56)	326 (11.5)
3rd - 40-50	885 (34.33)	884 (34.29)	930 (36.07)	931 (36.11)	857 (33.24)	936 (36.31)	173 (6.1)	173 (6.1)	176 (6.21)	175 (6.18)	164 (5.79)
4th - 55-65	774 (30.02)	772 (29.95)	803 (31.15)	811 (31.46)	797 (30.92)	808 (31.34)	88 (3.11)	88 (3.11)	91 (3.21)	87 (3.07)	151 (5.33)
Adherence criteria	2235 (86·7)*	2228 (86·42)*	712 (27·62)**	715 (27.73)**	518 (20.09)**	717 (27.81)**	1949 (68·77)*	1790 (63.16)*	72 (2.54**)	68 (2.4)**	53 (1.87)**

CAPTION: n= number; N=total number; %=percentage; Frequency of no maternal assessment within 65 minutes postpartum: E-MOTIVE care=310; usual care=622
~ blood pressure & pulse & uterine tone & vaginal blood flow; † Novel to E-MOTIVE care; * Clinical sign assessed at least once within 65 minutes; **Clinical sign assessed during all time increments within 65 minutes

Appendix 2: Ethics and permissions statement

This study has received ethics approval and permissions from the following entities:

United Kingdom:
University of Birmingham STEM committee (Reference number: ERN_19-1557E).
Kenya:
University of Nairobi: KNH/ERC/Mod&SAE/430;
Pharmacy and Poisons Board PPB/ECCT/20/06/06/2020(116),
National Commission for Science, Technology and Innovation Nacosti P/21/8437.
Nigeria:
National Health Research Ethics Committee of Nigeria (NHREC) (Reference number: NHREC/01/01/2007)-07/04/2022D).
South Africa:
Eastern Cape Department of Health (EC_202007_015);
University of Cape Town Human Research Ethics Committee (HREC; reference number: 091/2020),
Health Province of KwaZulu-Natal (NHRD reference number: KZ_202008_036),
University of the Witwatersrand Human Research Ethics Committee-Medical (reference number: M200241).
Tanzania:
Muhimbili University of Health and Allied Sciences (MUHAS) (reference number: DA.282/298/06/C/767);
National Institute for Medical Research (NIMR) (Reference number: NIMR/HQ/R.8a/Vol IX/3510).

The E-MOTIVE trial aimed to change health worker behaviours regarding PPH diagnosis and management. Hospital leadership at each site provided facility-level permission for the observations, while healthcare providers gave written informed consent before trial-specific training. Monitoring visits and observations of healthcare professionals managing vaginal births were conducted during baseline and intervention phases. Individual-level consent from women for these observations was not sought, as women were not the target of the intervention, were not interacted with for observation data collection, and no identifiable information on the observed women was collected or linked to them. The trial was conducted in accordance with the Declaration of Helsinki, CIOMS International Ethical Guidelines, and the Ottawa Statement for the Ethical Design and Conduct of Cluster Randomised Trials.

Appendix 3: Documentation

Observation guides

E-MOTIVE care - https://osf.io/7xmgc

Usual care - https://osf.io/m47pt

Blood loss monitoring chart

Nigeria, Kenya, and Tanzania - https://osf.io/j4uas

South Africa - https://osf.io/sy4h7
